# Address of Dr. Durkee

**Published:** 1856-08

**Authors:** 


### EXTRACT OF A SPEECH

Of Dr. Durkee, at the Annual Dinner of the Massachusetts Medical Society, May, 1856.

Mr. Chairman:—You have alluded to me as a lover and a student of microscopic anatomy. It is true that for some years I have been charmed with the beauties and the wonders which the microscope reveals in connection with our physical frame.

But I must tell you also, Mr. Chairman, that I have sometimes been shocked at the sight of some abnormal growth, which is now and then developed both in our material and psychological organization. There is one form of stern and hideous development appertaining to poor humanity which it is truly distressing to find. The product to which I allude is not mentioned in any strictly scientific work on Anatomy. And yet I will not pretend that I have discovered anything new. From what I have seen of the matter, and according to the best process of analytical anatomy to which I have been able to subject it, it appears to be of a benign character in the incipient stages of its growth, so far as one can judge both with the microscope and without it. But if allowed to remain in the system, and to progress in its tendencies, it is exceedingly apt to degenerate—and to assume a malignant aspect. From the few specimens I have seen, I have not been able to say that it has any fixed, any well defined, or uniform shape. It will be sufficiently truthful to say, on this point, that it is amorphous or polymorphous. Its elementary constituents possess great durability, and in this particular there is a seeming propriety in regarding it as osseous in its real character. It contains numerous winding canaliculi, and many sly and left-handed retreats, into which the individual can plunge, and from which he can emerge at will and cut up all manner of capers, which remind us of the snares and traps of Hiawatha.

Its properties are such that it will never bend, and it breaks with extreme difficulty. The fragments sometimes come together again with a violent and spasmodic force. I should have mentioned that it contains a large portion of earthy matter. It may be said to be "of the earth, earthy."

Although in the elegant and refined language of descriptive anatomy it can hardly be designated as bone, yet in the metaphorical diction of oriental poetry, as well as in the chaste and classical dialect of our red ancestors of the forest, it is eminently entitled to that name. This substance, by whatever epithet we call it, lies deeply buried in some of the soft tissues, especially the brain, which upon post mortem examination, is always found to be preternaturally soft and flat. The morbid growth in question has a great many salient angles, and is a very rough affair to handle. It is extraordinary, not only in its composition, but also in its effect upon the economy. It eats like a cancer, unless its action or the substance itself be removed, root and branch, by some adroit and philanthropic operation, that graces the annals of modern surgery; and its place be supplied by something more pure and genial in its attributes. Of one thing I am satisfied, and that is, that the operation for its removal should be performed at an early day. To this rule there is no exception; for if suffered to remain and exercise its dominion over the patient, it always proves to him and to those about him, what the foulest and blackest smut is to corn, what mildew is to wheat, and the curculio is to our choicest fruit bearers. Whatever be the position, or the ancestry, or the prospects, or the hopes, of the individual who carries it in his constitution, it strews his path with brambles by day, and gives him a pillar of thorns by night. It stings him like a scorpion; and rather than have it about him, he had better roll himself in the dust, like a dog that would rid himself of parasites. He is continually tossed upon a sea of troubles, and he poisons everything he touches. The effects upon some of the organs of sense are strange enough. The laws of vision seem to be broken, and no oculist is able to restore them. The eyes are injected with a fiery red, as in hydrophobia; and when he looks into a glass the demented man knows not himself. In fact his light is put under a bushel. The hearing is nearly gone. The poor wight is as deaf as the veriest adder. The voice of kindness, and persuasion, and reason, he connot hear; and to speak to him is like beating the East wind with a feather.

In the language of Shakspeare—

> " 'Tis a cause that hath no mean dependence
> Upon our joint and several dignities."

Without keeping you any longer in the dark, Mr. Chairman, let me tell you that I have in my mind's eye, the bone of contention. If there be such a bone, whenever and wherever found, let it be cut out by the deepest surgery: and, like the bones of Moses, let it be buried where no man can find it; and let its place be filled with the warm and vital current of brotherly love!

> " Bathe now in the streams before you,
> Wash the war paint from your faces,
> Wash the blood stains from your fingers,
> Bury your war clubs and your weapons,
> Break the red stone from the quarry,
> Mould and make them into peace-pipes ;—
> Take the reeds that grow beside you,
> Deck them with your brightest feather ;—
> Smoke the calumet together,
> And as brothers live forever."

